# The Evolving Role of Chlorthalidone and Hydrochlorothiazide as First-Line Treatments for Hypertensive Patients

**DOI:** 10.7759/cureus.63841

**Published:** 2024-07-04

**Authors:** Alan D Kaye, Sarah C Corley, Ellen Ingram, Peter P Issa, Logan T Roberts, Elisa E Neuchat, Matthew J Sharpe, Nicolette Doan, Olga Willett, Adam M Kaye, Sahar Shekoohi, Giustino Varrassi

**Affiliations:** 1 Departments of Anesthesiology and Pharmacology, Toxicology and Neurosciences, Louisiana State University Health Sciences Center, Shreveport, USA; 2 School of Medicine, Louisiana State University Health Sciences Center, New Orleans, USA; 3 School of Medicine, Louisiana State University Health Sciences Center, Shreveport, USA; 4 Department of Anesthesiology, Louisiana State University Health Sciences Center, Shreveport, USA; 5 Department of Pharmacy Practice, Thomas J. Long School of Pharmacy and Health Sciences, University of the Pacific, Stockton, USA; 6 Pain Medicine, Paolo Procacci Foundation, Rome, ITA

**Keywords:** hypertensive patients, first-line, hypertension, chlorthalidone, hydrochlorothiazide

## Abstract

Hypertension is attributable long-term to various negative health outcomes, including atherosclerotic cardiovascular disease and, more broadly, to cardiovascular events such as congestive heart disease, myocardial infarction, heart failure, and stroke. Effective hypertension treatment is essential to lower the risk of these outcomes. Treatment of hypertension includes both nonpharmacologic and, if necessary, pharmacologic interventions. The drug classes proven in trials to decrease the risk of cardiovascular disease events in cases with hypertension include angiotensin-converting enzyme inhibitors, angiotensin receptor blockers, thiazide diuretics, and calcium channel blockers. When considering thiazide diuretics as a first-line treatment, chlorthalidone (CTD) is currently recommended by the American College of Cardiology over hydrochlorothiazide (HCTZ). Previous studies have demonstrated that CTD is superior to HCTZ in preventing cardiovascular disease events. However, more recent studies have revealed that there is no significant difference in the results of patients treated with HCTZ versus those treated with CTD. Additionally, studies have revealed CTD has worse outcomes regarding side effects when compared to HCTZ. In this regard, it is essential to carefully consider which medication will best improve the outcomes of patients with hypertension while also causing few or easily manageable side effects.

## Introduction and background

Hypertension is a common problem worldwide, which is associated with various comorbidities such as cerebrovascular disease, cardiovascular disease, and renal disease [1]. In 2017, the American College of Cardiology estimated that approximately 116 million adults in the United States have hypertension, meaning almost half of Americans suffer from the disease [2]. There are many long-term negative health outcomes associated with hypertension. For instance, hypertension places a patient at an increased risk of atherosclerotic cardiovascular disease (CVD). Hypertension is also attributable to cardiovascular events, including congestive heart disease, myocardial infarction, heart failure, and stroke [1]. The main concern, then, when considering how to treat a patient with hypertension is preventing these outcomes. Treatment of hypertension includes both nonpharmacologic and, if necessary, pharmacologic interventions. Some nonpharmacologic interventions include weight loss, a Dietary Approach to Stop Hypertension (DASH) diet, and other dietary modifications. For pharmacologic interventions, four drug classes are proven in trials to reduce the risk of cardiovascular disease in patients with hypertension [1]. Those medication classes include angiotensin-converting enzyme (ACE) inhibitors, angiotensin receptor blockers, thiazide diuretics, and calcium channel blockers. Pharmacologic therapy for hypertension has been shown to decrease the relative risk for atherosclerotic CVD, and the amount of risk reduction is even more significant in those patients with higher CVD risk when compared to those with lower CVD risk [1]. When choosing one of these medications, there are many factors to consider, including effectiveness, side effects, and cost. When considering specifically thiazide diuretics, the American College of Cardiology recommends using chlorthalidone over hydrochlorothiazide (HCTZ) related to previous studies showing that chlorthalidone is superior to HCTZ in preventing cardiovascular disease events [3,4]. However, researchers recently have shown that there is no difference in outcomes but an increased risk of side effects in patients treated with chlorthalidone [5-7].

This narrative review, therefore, describes current hypertension practice guidelines, commonly used hypertension medications, medication side effects, and whether or not HCTZ should be used as first-line treatment over chlorthalidone.

## Review

Methods

This is a narrative review. The sources for this review were found by searching PubMed, Google Scholar, Medline, and ScienceDirect using the keywords hypertensive, patients, first-line, hypertension, chlorthalidone, and hydrochlorothiazide.

Current practice guidelines

The American Heart Association (AHA) and the European Society of Cardiology (ESC) hypertension guidelines focus on measuring blood pressure using accurate devices and separate measurements. Per AHA, the average value of two or more measurements should be done using the same device on at least two visits. ESC suggests three measurements with an additional measurement only if two readings vary greater than or equal to 10 mmHg. Per the current practice guidelines from the American Cardiology Association, hypertension is generally categorized as stage 1 and stage 2 [1]. Stage 1 hypertension means a systolic blood pressure of 130-139 mmHg or a diastolic blood pressure of 80-89 mmHg. In stage 2 hypertension, the systolic blood pressure is higher than or equal to 160 mmHg or diastolic blood pressure is greater than or equal to 100 mmHg. The blood pressure used for diagnosis should be based on an average of 14 measurements over seven days. It is also recommended before diagnosis to have blood pressure measurements from outside a clinical setting [1]. Some other nonpharmacologic interventions recommended for hypertension management are a Dietary Approach to Stop Hypertension (DASH) diet, dietary sodium reduction, potassium supplementation unless contraindicated, and increased physical activity [1]. If a patient with stage 1 hypertension has less than 10 percent 10-year atherosclerotic cardiovascular disease risk, it is currently recommended to first attempt nonpharmacologic therapy. However, for those with a 10 percent or higher 10-year atherosclerotic cardiovascular disease risk, a patient with stage 1 blood pressure is recommended to start both non-pharmacologic and anti-hypertensive medication to achieve secondary prevention of cardiovascular disease (CVD). If a patient has stage 2 hypertension, regardless of atherosclerotic cardiovascular disease risk, the patient should begin both nonpharmacologic interventions and anti-hypertensive medications. Furthermore, two medications of different classes should be considered if a patient has stage 2 hypertension in addition to having systolic blood pressure and diastolic blood pressure elevated 20 and 10 mmHg, respectively, over the goals [1]. Regarding which anti-hypertensive medication to give a patient, the chief goal of treatment is to reduce the incidence of cardiovascular disease events. The treatments with proven efficacy are thiazide diuretics, calcium channel blockers (CCBs), angiotensin-converting enzyme (ACE) inhibitors, and angiotensin receptor blockers (ARBs) [1].

Anti-hypertensive medication options

Thiazide Diuretics

Hydrochlorothiazide (HCTZ) and chlorthalidone (CTD) are two common diuretics in the thiazide class of drugs. Specifically, these drugs act at the distal convoluted tubule of the nephron to inhibit an apical-surface sodium-chloride symporter. In diluting the capacity of the distal convoluted tubule and nephron as a whole, HCTZ and CTD allow functional diuresis, lowering the blood pressure. Globally available with implications in medicine for more than half a century, HCTZ has been approved by the Food and Drug Administration (FDA) for managing high blood pressure (as either a sole agent or adjunct), edema related to renal dysfunction, and edema associated with congestive heart failure and/or hepatic cirrhosis [8]. Standard HCTZ regimens for the management of hypertension vary from person to person and disease severity but typically include a 12.5 to 50 mg dose taken orally, once daily, given its 6- to 15-hour half-life [8]. Numerous works have demonstrated consistent reductions in systolic (5-7 mmHg) as well as diastolic blood pressure (4-5 mmHg) with low-dose (12.5 or 25 mg) daily HCTZ and even larger reductions at higher doses (e.g., 50 mg). The other more commonly utilized thiazide diuretic is chlorthalidone. While both medications are within the thiazide class, differences stem from their pharmacodynamic and pharmacokinetic properties, including the peak of action and duration of action (HCTZ: early peak, relatively long-lasting vs. CTD: very long-lasting (t1/2 = 40-60 hours) with a drug reservoir given its high volume of blood distribution) [9,10].

Other commonly used anti-hypertensive medications

While thiazide diuretics are a standard first-line option for managing hypertension, other first-line, efficacious classes of medications exist, including CCBs, ACE inhibitors, ARBs, and beta-blockers.

Calcium Channel Blockers

CCBs are first-line stand-alone or combination medications for treating high blood pressure in patients without significant renal involvement (ACE inhibitors and ARBs are renoprotective) [1,11]. As the name suggests, CCBs block voltage-gated L-type calcium channels on smooth and/or cardiac muscle cells. CCBs that act exclusively on smooth muscle cells include dihydropyridines (DHPs), and those with dual action on both cardiomyocytes and smooth muscle cells are aptly named non-DHPs. DHPs are potent peripherally acting vasodilators and include medications that end in -dipine, such as amlodipine. In a randomized controlled trial, amlodipine was found to similarly reduce the risk of cardiovascular morbidity as HCTZ in obese patients (p=0.32) [12]. As compared to the common beta-blocker atenolol, amlodipine prevented more major cardiovascular events (hazard ratio (HR): 0.84, 95%CI: 0.78-0.90, p<0.0001) and further decreased the risk of all-cause mortality (HR: 0.89, 95%CI: 0.81-0.99, p=0.025) in hypertensive patients [13]. Non-DHPs such as verapamil have been shown to reduce blood pressure as well effectively. However, their use is less common and is contraindicated in cases with congestive heart failure with reduced ejection fraction, given the risk of cardiac depression/block.

Angiotensin-Converting Enzyme Inhibitors and Angiotensin Receptor Blockers

Given their renoprotective effects, ACE inhibitors and ARBs are first-line options for patients with high blood pressure and chronic kidney disease or heart failure [11]. Thiazide diuretics and CCBs are generally more efficacious than ACE inhibitors in managing hypertension [1,14]. ACE inhibitors and ARBs work on the same physiological pathway, the renin-angiotensin-aldosterone system, with ARBs working further downstream, which leads to minor differences between the two classes [14]. Notably, the two classes of medications are not recommended to be used simultaneously. In addition, one notable difference between ACE inhibitors and ARBs is that the former are relatively contraindicated in managing patients with chronic cough, given that their use may induce/exacerbate a cough by preventing bradykinin degradation [14].

Beta-Blockers

Beta-blockers are not typically indicated for the management of high blood pressure, except in specific conditions such as myocardial infarction or heart failure, and are consequently a second-line management option [15,16]. While the beta-blocker use can reduce the risk of cardiovascular morbidity in patients with hypertension, numerous cautions exist and limit their general use. For example, beta-blockers are less effective in elderly cases (>65 years), are associated with an increased risk of stroke, may potentially mask hypoglycemia (contraindicated in diabetic patients), and may allow unopposed bronchoconstriction (contraindicated in asthma patients) [17,18].

Adverse effects of thiazide diuretics compared to the adverse effects of ACE inhibitors and CCBs

Thiazide diuretics have a relatively low side effect profile, as do ACE inhibitors and CCBs. All of them can be used as the first-line medications to treat essential hypertension. However, it is important to compare their adverse effects as well as the patient's preference to determine the best medication to prescribe to a hypertensive patient. In Table 1, we compare the adverse effects of these pharmaceuticals. ACE inhibitors can cause a dry cough secondary to increased bradykinin concentrations; however, angiotensin receptor blockers have a lower incidence of cough production [19,20]. ACE inhibitors can also cause angioedema, which can be life-threatening and is a reason to discontinue ACE inhibitors forever [19,21]. CCBs are a well-known cause of peripheral edema. They preferentially dilate pre-capillary blood vessels, resulting in blood pooling in the lower extremities [22]. The thiazide diuretics can cause numerous electrolyte abnormalities due to their effects at the nephron level. One of their major adverse effects is hypokalemia and hyponatremia [23]. If this becomes a problem, one may consider switching to a potassium-sparing diuretic such as spironolactone.

**Table 1:** Adverse effects of ACE inhibitors, CCBs, and thiazide diuretics. Sources: [24-27] ACE: angiotensin-converting enzyme CCBs: calcium channel blockers

Medication	Adverse effects
ACE inhibitors	First-dose hypotension, azotemia, cough, fatigue, hyperkalemia, angioedema, teratogenicity
Calcium channel blockers	Constipation, bradycardia, lightheadedness, flushing headaches, peripheral edema
Thiazide diuretics	Hypokalemia, hyponatremia, hyperuricemia, elevated plasma glucose, elevated plasma cholesterol

Comparison of efficacy and side effects of chlorthalidone and hydrochlorothiazide

Within the thiazide diuretic drug class, two commonly used medications are hydrochlorothiazide and chlorthalidone. In the recommendations from the American Cardiology Association, chlorthalidone is preferred to hydrochlorothiazide for various reasons, including that chlorthalidone has a longer half-life [1]. Chlorthalidone also has ample evidence to prove its efficacy in decreasing cardiovascular-related mortality events [1]. There has been much debate as to which formulation of thiazide diuretic is better for patients. In Table 2, we describe multiple studies that compare hydrochlorothiazide to chlorthalidone. *How were these studies identified? Was there a timeline or a search strategy?* Studies have compared the two medications that showed chlorthalidone to be superior in preventing cardiovascular events [3,4]. According to some more recent studies, chlorthalidone was better at lowering blood pressure, but it carried an increased risk of electrolyte abnormalities and did not decrease mortality from cardiovascular-related events when compared to hydrochlorothiazide [5,6,9].

**Table 2:** List of recent and earlier works comparing the efficacy and side effects of hydrochlorothiazide (HCTZ) to chlorthalidone (CTD) for the management of hypertension. AA = African American, CI = confidence interval, EA = European American, eGFR = estimated glomerular filtration rate; HR = hazard ratio, LDL = low-density lipoprotein, RR = relative risks, SBP = systolic blood pressure

Author (Year)	Groups Studied and Intervention		Results and Findings	Conclusions
Dorsch et al. (2011): “Chlorthalidone reduces cardiovascular events compared with hydrochlorothiazide: a retrospective cohort analysis” [4]		A retrospective cohort analysis of 12,866 men aged 35-57 years enrolled from 1973	CTD has a lower cardiovascular event risk than HCTZ (adjusted HR 0.51%, 95% CI: 0.43-0.61). CTD also seemed to lead to more reduction in SBP than HCTZ. CTD resulted in increased levels of uric acid (p<0.0001) and lower levels of potassium (p=0.0003) when compared to HCTZ. CTD had lower LDL cholesterol when compared to HCTZ (p<0.0001)	CTD had decreased cardiovascular events when compared to no treatment and also HCTZ. CTD had worse side effects than HCTZ when considering uric acid levels and effects on potassium levels.
Roush et al., (2012): “Chlorthalidone compared with hydrochlorothiazide in reducing cardiovascular events: systematic review and network meta-analyses” [3]		Systematic review of nine randomized clinical trials and isolating the data from the arms analyzing CTD and HCTZ	CTD led to a risk reduction 23% (95% CI: 2-39) of congestive heart failure when compared to HCTZ. CTD also has better risk reduction of all cardiovascular events than HCTZ (RR 21%, 95% CI: 12-28). The reported number needed to treat with CTD over HCTZ to prevent one cardiovascular event is 27.	CTD, when compared to HCTZ, leads to a better risk reduction of both congestive heart failure and general cardiovascular events.
Hripcsak et al. (2020): “Comparison of cardiovascular and safety outcomes of chlorthalidone vs hydrochlorothiazide to treat hypertension” [6]		730,255 individuals first-ever treated for hypertension by HCTZ or CTD	Similar efficacy (HR: 1.00, 95%CI: 0.85-1.17). Increased risk of adverse events with CTD including hypokalemia (HR: 2.72, 95%CI: 2.38-3.12), hyponatremia (HR: 1.31, 95%CI: 1.16-1.47), and acute renal failure (HR:1.37, 95% CI, 1.15-1.63).	No significant benefit with CTD as compared to HCTZ, though it increased the risk of renal and electrolyte abnormality.
Edwards et al. (2021): "Comparison of clinical outcomes and safety associated with chlorthalidone vs hydrochlorothiazide in older adults with varying levels of kidney function” [7]		Patients with hypertension treated with either HCTZ (n=9,786) or CTD (n=2,936)	Use of CTD as compared to HCTZ: Increased risk of eGFR decline (HR 1.24, 95%CI: 1.13-1.36) and cardiovascular morbidity (HR 1.12, 95%CI: 1.04-1.22). In eGFR ≥ 60, increased risk of hypokalemia (HR 1.86, 95%CI: 1.67-2.08). In eGFR = 45-59, increased risk of hypokalemia (HR 1.57, 95%CI: 1.25–1.96).	There is little to no evidence to support the use of CTD as compared to HCTZ.
Ishani et al. (2022): “Chlorthalidone vs. hydrochlorothiazide for hypertension–cardiovascular events” [5]		13,523 patients on HCTZ were randomly assigned to maintain HCTZ or switch to CTD	Similar rates of major cardiovascular events or non-cancer related deaths (p=0.45). Higher rates of hypokalemia with CTD use (HCTZ: 4.4% vs. CTD: 4.4%, p<0.001).	No decreased risk in major cardiovascular events in patients managed by CTD as compared to HCTZ. Little-to-no merit in transitioning patients from HCTZ to CTD.
Dineva (2021): “Network meta-analysis of efficacy and safety of chlorthalidone and hydrochlorothiazide in hypertensive patients” [9]		Meta-analysis of 28 studies indirectly comparing and 9 studies directly comparing HCTZ and chlorthalidone	When analyzing indirect comparison, the difference in reduction of diastolic blood pressure between CTD and HCTZ was not significant (95%CI, -2.02 to 0.84), but the difference in reduction of systolic blood pressure between CTD and HCTZ was (-4.74) and significant (95%CI, −7.20 to −2.28). When analyzing combined indirect and direct data, the difference in reduction of diastolic blood pressure between CTD and HCTZ was (−0.67) and not significant (95%CI, −1.92 to 0.57), and the difference in reduction of systolic blood pressure between CTD and HCTZ was (-2.35) and not significant (95%CI, −5.52 to 0.83).	HCTZ and chlorthalidone can be used interchangeably as blood pressure medications, but chlorthalidone has slightly higher efficacy with regard to lowering systolic blood pressure.
Chekka et al. (2022): “Pairwise comparison of hydrochlorothiazide and chlorthalidone responses among hypertensive patients” [28]		50 patients pairwise comparison treatment by HCTZ and CTD	In EA patients, mean reduction on HCTZ vs. CTD was 8/5 vs. 16/8 mmHg (p=0.002). In AA patients, mean reduction on HCTZ vs. CTD was 11/8 versus 20/11 mmHg (p=0.22).	CTD may be superior to HCTZ in a majority of EA and a minority of AA patients in decreasing systolic and diastolic blood pressure.

Though hydrochlorothiazide is the most commonly prescribed thiazide diuretic, the recent (2017) American College of Cardiology (ACC) and AHA guidelines recommend the use of chlorthalidone as the thiazide of choice given its reduction in cardiovascular disease and longer half-life [1]. This change in recommendation was secondary to a handful of works in the early 2010s, which suggested that chlorthalidone was superior from efficacy and safety standpoints [3,4]. However, recent landmark works have reported superior or non-inferior efficacy and safety outcomes in patients prescribed hydrochlorothiazide [5-7]. For example, Hripcsak et al. analyzed over 730,000 patients taking either hydrochlorothiazide or chlorthalidone and reported similar efficacy (HR: 1.00, 95%CI: 0.85-1.17) but increased risk of adverse events with chlorthalidone, including hypokalemia (HR: 2.72, 95%CI: 2.38-3.12), hyponatremia (HR: 1.31, 95%CI: 1.16-1.47), and acute renal failure (HR:1.37, 95% CI, 1.15-1.63) [6]. Similarly, in 2022, Ishani et al. published the findings of their randomized controlled trial, including 13,524 patients older than 65 years initially on HCTZ who were randomized to maintain HCTZ or switch to CTD [5]. The authors reported similar rates of major cardiovascular events or non-cancer-related deaths (p=0.45) but higher rates of hypokalemia with chlorthalidone use (HCTZ: 4.4% vs. CTD: 4.4%, p<0.001), concluding that little-to-no differences between the two medications may not merit the transition to CTD [5]. While the recent literature tends to suggest hydrochlorothiazide as the superior or non-inferior thiazide, future investigations including prospective multi-institutional works are recommended to corroborate and expand on any clinical differences between hydrochlorothiazide and chlorthalidone.

Discussion

Related to the availability of various efficacious blood-pressure-lowering medications, choosing the best regimen often requires subjective clinical judgment and patient-specific factors. Since elevated blood pressure is an indirect variable used to assess long-term cardiovascular disease risk, it is best evaluated in combination with other factors to gauge the overall cardiovascular risk of a patient. Non-pharmacological management may be considered low risk, but the efficacy of this management is largely determined by the patient’s ability to incorporate exercise routines and dietary changes. In higher-risk patients, a lower threshold for initiation of pharmacological management is recommended. For these patients, other cardiovascular risk factors are considered to support earlier initiation and more aggressive blood-pressure-lowering regimens. When choosing between the recommended first-line agents, patient-specific factors and side-effect profiles should be considered to improve outcomes and compliance. For example, when considering thiazide diuretics, the increased urinary frequency could bother some patients, especially those with a decreased ability to ambulate. Thiazide should also be used carefully in patients with gout due to the side effects of hyperuricemia. Calcium channel blockers are known to cause peripheral edema and should not be utilized in patients with heart failure with reduced ejection fraction. While all the classes mentioned are effective in patients with diabetes, ACE inhibitors and ARBs should be considered more in patients with albuminuria. Once an initial regimen has been selected, side effect monitoring should be continued throughout treatment, and alterations should be made if warranted. It is essential to be aware of side effects commonly associated with each drug class so that early recognition and appropriate action can be taken to minimize these occurrences and maximize patient compliance. Furthermore, recommended dosing ranges should be followed for each drug to maximize therapeutic effect, reduce exposure and unnecessary side effects, and recognize when a second agent is initiated. 

## Conclusions

Treatment of hypertension includes both nonpharmacologic and, if necessary, pharmacologic interventions. The drug classes proven in trials to decrease the risk of cardiovascular disease events in cases with hypertension include angiotensin-converting enzyme inhibitors, angiotensin receptor blockers, thiazide diuretics, and calcium channel blockers. More research is needed to establish which thiazide diuretic is a better choice to treat hypertension. Regardless, when used appropriately in the recommended patient population, both thiazide diuretics (chlorthalidone and hydrochlorothiazide) remain a suitable first-line option for the management of high blood pressure due to their wide therapeutic index, well-documented effectiveness, and favorable tolerability.

## References

[1] Whelton PK, Carey RM, Aronow WS (2018). 2017 ACC/AHA/AAPA/ABC/ACPM/AGS/APhA/ASH/ASPC/NMA/PCNA Guideline for the Prevention, Detection, Evaluation, and Management of High Blood Pressure in Adults: Executive Summary: A Report of the American College of Cardiology/American Heart Association Task Force on Clinical Practice Guidelines. Hypertension.

[2] (2017). Hypertension Cascade: Hypertension Prevalence, Treatment and Control Estimates Among U.S. Adults Aged 18 Years and Older Applying the Criteria from the American College of Cardiology and American Heart Association’s 2017 Hypertension Guideline—NHANES 2015-2018. American College of Cardiology and American Heart Association’s.

[3] Roush GC, Holford TR, Guddati AK (2012). Chlorthalidone compared with hydrochlorothiazide in reducing cardiovascular events: systematic review and network meta-analyses. Hypertension.

[4] Dorsch MP, Gillespie BW, Erickson SR, Bleske BE, Weder AB (2011). Chlorthalidone reduces cardiovascular events compared with hydrochlorothiazide: a retrospective cohort analysis. Hypertension.

[5] Ishani A, Cushman WC, Leatherman SM (2022). Chlorthalidone vs. hydrochlorothiazide for hypertension-cardiovascular events. N Engl J Med.

[6] Hripcsak G, Suchard MA, Shea S (2020). Comparison of cardiovascular and safety outcomes of chlorthalidone vs hydrochlorothiazide to treat hypertension. JAMA Intern Med.

[7] Edwards C, Hundemer GL, Petrcich W (2021). Comparison of clinical outcomes and safety associated with chlorthalidone vs hydrochlorothiazide in older adults with varying levels of kidney function. JAMA Netw Open.

[8] Herman LL, Bashir K (2021). Hydrochlorothiazide. StatPearls.

[9] Dineva S, Uzunova K, Pavlova V, Filipova E, Kalinov K, Vekov T (2021). Network meta-analysis of efficacy and safety of chlorthalidone and hydrochlorothiazide in hypertensive patients. Blood Press Monit.

[10] Roush GC, Buddharaju V, Ernst ME, Holford TR (2013). Chlorthalidone: mechanisms of action and effect on cardiovascular events. Curr Hypertens Rep.

[11] Xie X, Liu Y, Perkovic V (2016). Renin-angiotensin system inhibitors and kidney and cardiovascular outcomes in patients with CKD: a bayesian network meta-analysis of randomized clinical trials. Am J Kidney Dis.

[12] Weber MA, Jamerson K, Bakris GL (2013). Effects of body size and hypertension treatments on cardiovascular event rates: subanalysis of the ACCOMPLISH randomised controlled trial. Lancet.

[13] Dahlöf B, Sever PS, Poulter NR (2005). Prevention of cardiovascular events with an antihypertensive regimen of amlodipine adding perindopril as required versus atenolol adding bendroflumethiazide as required, in the Anglo-Scandinavian Cardiac Outcomes Trial-Blood Pressure Lowering Arm (ASCOT-BPLA): a multicentre randomised controlled trial. Lancet.

[14] Li EC, Heran BS, Wright JM (2014). Angiotensin converting enzyme (ACE) inhibitors versus angiotensin receptor blockers for primary hypertension. Cochrane Database Syst Rev.

[15] Khan N, McAlister FA (2006). Re-examining the efficacy of beta-blockers for the treatment of hypertension: a meta-analysis. CMAJ.

[16] Carey RM, Moran AE, Whelton PK (2022). Treatment of hypertension: a review. JAMA.

[17] Thomopoulos C, Parati G, Zanchetti A (2018). Effects of blood pressure-lowering treatment on cardiovascular outcomes and mortality: 14 - effects of different classes of antihypertensive drugs in older and younger patients: overview and meta-analysis. J Hypertens.

[18] Wiysonge CS, Bradley H, Mayosi BM, Maroney R, Mbewu A, Opie LH, Volmink J (2007). Beta-blockers for hypertension. Cochrane Database Syst Rev.

[19] Morimoto T, Gandhi TK, Fiskio JM (2004). An evaluation of risk factors for adverse drug events associated with angiotensin-converting enzyme inhibitors. J Eval Clin Pract.

[20] Chen R, Suchard MA, Krumholz HM (2021). Comparative first-line effectiveness and safety of ACE (angiotensin-converting enzyme) inhibitors and angiotensin receptor blockers: a multinational cohort study. Hypertension.

[21] Beltrami L, Zingale LC, Carugo S, Cicardi M (2006). Angiotensin-converting enzyme inhibitor-related angioedema: how to deal with it. Expert Opin Drug Saf.

[22] Gustafsson D (1987). Microvascular mechanisms involved in calcium antagonist edema formation. J Cardiovasc Pharmacol.

[23] Franse LV, Pahor M, Di Bari M, Somes GW, Cushman WC, Applegate WB (2000). Hypokalemia associated with diuretic use and cardiovascular events in the Systolic Hypertension in the Elderly Program. Hypertension.

[24] Kostis JB, Shelton B, Gosselin G (1996). Adverse effects of enalapril in the Studies of Left Ventricular Dysfunction (SOLVD). SOLVD Investigators. Am Heart J.

[25] McKeever RG, Hamilton RJ (2023). Calcium channel blockers. StatPearls.

[26] Leung AA, Wright A, Pazo V, Karson A, Bates DW (2011). Risk of thiazide-induced hyponatremia in patients with hypertension. Am J Med.

[27] Carlsen JE, Køber L, Torp-Pedersen C, Johansen P (1990). Relation between dose of bendrofluazide, antihypertensive effect, and adverse biochemical effects. BMJ.

[28] Chekka LM, Cooper-DeHoff RM, Gums JG, Chapman AB, Johnson JA (2022). Pairwise comparison of hydrochlorothiazide and chlorthalidone responses among hypertensive patients. Clin Transl Sci.

